# A Review of the Impact of Selected Anthropogenic Chemicals from the Group of Endocrine Disruptors on Human Health

**DOI:** 10.3390/toxics9070146

**Published:** 2021-06-24

**Authors:** Katarzyna Goralczyk

**Affiliations:** Institute of Biology Science in Warsaw, University of Cardinal Stefan Wyszynski, Wóycickiego 1/3, 01-938 Warsaw, Poland; k.goralczyk@uksw.edu.pl

**Keywords:** xenoestrogens, endocrine-disrupting chemicals, disease of civilization

## Abstract

Background: The aim of the study was to review data on the impact of anthropogenic chemicals (endocrine disruptors) on various diseases, which, consequently, may facilitate their prevention and be used as a tool for managing public healthcare. Every day, humans are exposed to chemicals, including xenoestrogens, which are similar to female hormones. Methods: This manuscript was prepared based on a meta-analysis of research on the impacts of selected EDCs on human health. Results: Special attention should be paid to bisphenol A (BPA), benzo-α-pyrene, and phthalates due to their proven endocrine activity and presence in our daily lives. Xenoestrogens are absorbed by human organisms through the digestive system since they can migrate to food from food packages and drinks as well as from plastic products used daily. The presence of these chemicals in human organisms is considered a potential cause for some diseases commonly referred to as ‘diseases of civilization’. Conclusions: The biomonitoring of xenoestrogens, which are chemicals with unfavorable impacts on human health, is a crucial tool for assessing the risk from the pollution of the environment. The novelty is a holistic approach to assessing the occurrence of risk factors for civilization diseases.

## 1. Introduction

Every day, humans are exposed to natural and synthetic chemical compounds, including xenoestrogens, which are similar to female hormones. These exogenous compounds may interact with the human hormonal system, leading to homeostasis disorders by modulating both estrogenic activities and androgenic activity itself [1].

The presence of anthropogenic endocrine disruptors (ECDs) in various elements of the environment is a direct or indirect threat to humans, as indicated by biomonitoring studies on the systemic distribution of biomarkers of human exposure to this group of compounds. ECDs are present in tissues, human body fluids, and breast milk, as well as in food, despite the introduction of restrictions on their use. The biomonitoring of compounds that adversely affect human health is an indispensable tool for assessing the risks related to environmental pollution while providing a substantial basis for risk management. Tracking trends regarding the levels of these compounds in various elements of the environment, including humans, is particularly important due to the toxicological properties of ECDs. The results of biomonitoring are an important element used in risk assessment, along with toxicological data, to enable the determination of the point of departure (PoD) when determining hazard profiles (HQ), or for preparing statistical models that, based on population data (age and gender) and the levels of biomarkers for selected chemical groups, can be used to determine the total concentrations of specific environmental pollutants in the human body. For a biomonitoring-based risk assessment, PoD can be defined as an internal exposure–response point that marks the threshold above which the incidence begins to increase. This approach also considers the fact that the internal exposure, expressed as serum concentrations above PoD, as being of concern. This is especially important in the case of xenoestrogens, for which a diverse geographical distribution and tissue distribution and different rates of release of individual compounds from the human body depending on age and sex have been demonstrated. This direction of research, which uses a holistic approach to describe the relationship between simultaneous exposure to a multitude of different risk factors and the body’s response, is a major public health challenge [2,3,4,5,6].

An essential and main area of interest for various research centers is the monitoring of risk markers of substances that tend to accumulate in humans and to determine their impact on health in different human populations. However, researchers are less interested in those xenobiotics that do not exhibit bioaccumulation properties. Xenoestrogens, which are classified as endocrine-disrupting chemicals (EDCs), have been of special interest for many years and are a subject of growing concern, especially regarding their potential biological risks, including the internal risk that comes from releasing deposits formed in the human organism. According to the WHO Classification, EDCs include esters of phthalic acid, bisphenols, polycyclic aromatic hydrocarbons (PAHs), polychlorinated biphenyls (PCBs), perfluoroalkylated substances (PFASs), bromoorganic flame retardants (FRs, e.g., polybrominated diphenyl ethers (PBDEs)), tributyltin (TBT), and parabens [1,2,7,8,9]. Among them are both persistent and less-persistent compounds. There is a lot of information in scientific publications about the impacts of POPs, such as PCBs, PBDEs, and PFASs, on human health. However, there are fewer studies on less-persistent compounds. Therefore, in this study, special attention was paid to the less-persistent EDCs, such as bisphenol A (BPA), benzo-α-pyrene (a representative of PAHs), and parabens (PHBs), due to their frequent use in various areas of daily human life.

Xenoestrogens are absorbed by humans, mainly through the digestive system, since they can migrate to food from food packages and drinks as well as from plastic products used daily [1,10]. For humans, the main source of such chemicals is food, mainly processed and packaged food. Their absorption from cosmetics and household chemicals should also be noted through direct dermal contact. Humans can take up EDCs, or precursors of EDCs, also by inhaling them, e.g., with cosmetic products. Human exposure varies considerably and depends on individual habits (e.g., food choices) and the locations where people work and live [1,7,8,10,11]. We spend about 90% of our lives in enclosed spaces. Sources of human exposure to EDCs have also been identified in indoor environments, such as homes and the workplace. Materials for building construction, furnishings, carpets, textiles, etc., are present in indoor environments. These lead to common classes of chemicals, such as formaldehyde, pesticides, phthalates, PCBs, brominated flame retardants, and parabens, being found in indoor air and dust [12,13,14,15,16,17].

## 2. Materials and Methods

The material was prepared based on a meta-analysis of research on the impact of selected EDCs on the health of a specific group of people. The review was limited to research published in peer-reviewed journals or other peer-reviewed studies as per the checklist of the Preferred Reporting Items for Systematic Reviews and Meta-Analyses (PRISMA) guidelines [18]. Data were collected by the manuscript author by systematically searching electronic databases of scientific publications, such as PubMed and Web of Science. Studies were included in this systematic review when the following criteria were met: (1) prospective cohort; (2) definitions of EDCs were in accordance with WHO definition; (3) concentration of chemicals was measured using biological specimens; (4) risk estimates including risk for described population (age, gender, place of residence). When selecting and rejecting publications, it was considered that the presented data were representative, i.e., the amount of data from individual continents was comparable. It was particularly important when selecting biomonitoring data.

## 3. Results

### 3.1. Toxic Mechanism of EDCs

#### 3.1.1. Humans

It has been proven that the mechanism of EDCs’ toxicity is based on their mimicry of endogenous hormones. The structural similarity of xenoestrogens to steroid sex hormones results in their easy association with estrogen and androgen receptors through competitive binding (a ligand with a chemical structure similar to estradiol’s associates with an estrogen receptor and activates it). The mechanism by which a chemical disrupts hormone action has a very large impact on the pattern of effects one would expect to observe. Generally, there are two pathways by which a chemical could disrupt hormone action: a direct action on a hormone receptor protein complex, or a direct action on a specific protein that controls some aspect of controlling hormone delivery to the right place at the right time. This could be a protein that is involved in hormone production (e.g., aromatase), an important transporter (e.g., sodium/iodide symporter), or a carrier protein (e.g., cortisol binding protein). EDCs act as antagonists of hormones. They may block estrogen receptors or, via the receptor, inhibit the activity of endogenous hormones. Xenoestrogens have multiple mechanisms of action, interact with different receptors, and affect the entire hormonal system. These involve the aryl hydrocarbon receptor (AhR), which regulates the expression of several genes, including the cytochrome P450 (CYP-1) gene family members and glutathione S-transferase M, which mediates many of the responses to environmental toxic chemicals. They influence synthesis, decomposition, or elimination from the system as well as hormone bio-accessibility; for example, they may directly influence gene expression by limiting the concentrations of protein-binding sex hormones [19,20,21,22]. Due to their similar influence on humans—that is, their sex-hormone-imitative effects on estrogen receptors as well as their similar toxicological properties and wide uses in different areas of life, both as a group or individually—xenoestrogens are the subject of various research studies with the aims of explaining their range of impacts on the human organism, conducting risk assessments, and consequently, determining their influences on health [6,23,24,25,26,27,28,29]. Table 1 presents the concentrations of selected EDCs in human biological materials. The data were selected to be representative both for a given geographic region and for a given country.

The presence of these chemicals in the human organism is considered a potential cause of some diseases, commonly referred to as ‘diseases of civilization’. The notion of ‘diseases of civilization’ is used to describe certain ailments whose etiology is difficult to explain based on knowledge about the functioning of the body and its metabolism. Only studies at the cellular level and biochemical changes have shed light on the causes of some diseases described as diseases of civilization (cancers, cardiovascular and respiratory diseases, obesity, psychomotor disorders in children, and malformations or neurodegenerative diseases that have increased in frequency). For example, over the past 20 years, the incidence of mental and behavioral disorders has increased by more than 37% (Parkinson’s disease has increased by 75%, Alzheimer’s disease has doubled, autism has increased by 30%, and attention deficit hyperactivity disorder (ADHD) has increased by 16%) [1,30]. Recent studies on the impact of EDC on the functioning of the human body focus on changes in physiology, behavior, and biochemical mechanisms in connection with the circadian cycle. Extensive bidirectional crosstalk exists between circadian and endocrine systems, and circadian rhythmicity is present at all levels of endocrine control, from synthesis and release of hormones to sensitivity of target tissues to hormone action. In mammals, a range of hormones directly alters clock gene expression and circadian physiology via nuclear receptor (NR) binding and subsequent genomic action, modulating physiological processes such as nutrient and energy metabolism, stress response, reproductive physiology, and circadian behavioral rhythms. Because EDCs mimic the action of hormones in the body and the potential of these compounds to disrupt the circadian rhythm is still insufficiently characterized, hence the new focus of research on this aspect. These studies concern: dysregulated core clock and circadian rhythm network gene expression in brain and peripheral organs, and altered circadian reproductive, behavioral, and metabolic rhythms. Circadian impacts occurred in parallel to endocrine and metabolic alterations such as impaired fertility and dysregulated metabolic and energetic homeostasis [32,33].

#### 3.1.2. Sources of EDCs

Numerous research studies, as shown in Table 2 and Table 3, have provided evidence that the main sources through which human beings come into contact with this type of chemical are processed and packaged foods as well as food contact materials (FCMs). This mainly concerns bisphenol A and PAHs [34,35,36,37,38]. Table 2 and Table 3 present the concentrations of selected EDCs in foods, food contact materials, and selected daily use products.

By considering the occurrence of selected EDCs in the environment, their wide use in different areas, and the biomonitoring results, it was possible to link the roles of these chemicals to the process of the development of various diseases that have shown a growing incidence in recent years.

The aim of this paper was to review data on the impacts of selected anthropogenic chemicals from the group of endocrine disruptors on various diseases, including ‘diseases of civilization’. These chemicals may facilitate the prevention of these diseases and be used as tools in managing public healthcare.

## 4. Discussion

### 4.1. Characteristics and Effects of Chosen Environmental Pollutants

Phthalates are esters of phthalic acid that belong to a group of chemicals that are widely used as emollients in plastic production, e.g., in the production of polyvinyl chloride (PCV). They are present in medical equipment (e.g., intravenous tubes, blood bags, and masks), building materials (e.g., insulating cables, pipes, and wallpaper), car equipment, toys, and home equipment and are used as additives in the textile industry, as carriers in pesticide formulations, as coatings in time-release pharmaceuticals, as industrial solvents, and as lubricants. Phthalates are also added to cosmetics (perfumes, sprays, paints, and fragrances) and household chemicals [1,40,42,43]. The ones used most widely phthalates are benzylbutyl phthalate (BBP), di-n-butyl phthalate (DBP), di-2(2-ethylhexyl) phthalate (DEHP), and nonylphenol phthalate (NP). Their wide use in various areas results in humans being exposed to these chemicals through different sources throughout their lifetimes. The main sources of phthalates are foods, drinking water, and house dust [8,35,38]. The presence of phthalates in the human organism (see Table 1) causes disorders of hormonal homeostasis, which negatively influences health. Based on numerous biomonitoring research studies, it has been proven that phthalates contribute to thyroid disorders, asthma, and allergies due to their presence in household dust. Phthalates may interfere with the thyroid gland, e.g., within the conduction of signals regarding the synthesis, transport, and level of TSH circulating in the serum. The presence of phthalates in the body can also negatively affect free and total T3 levels in children. These disorders may also affect the courses of biochemical processes, i.e., the proper working of various organs [26,44,45]. Moreover, phthalates influence the reproductive systems of both men and women. In men, exposure to phthalates results in disorders of cell differentiation and the development of androgen-dependent tissues and gonadal disorders. Furthermore, following risk exposure during the growth period, gonadal dysgenesis, and testicular and epididymis reduction as well as pathological and biochemical changes in the testicles, necrosis of the seminiferous tubule epithelium, lower sexual reproduction as a consequence of changes in the structure and functioning of the epididymis, and reductions in sperm quantity and quality can occur, which may lead to male infertility [1,46]. In women, phthalates may influence the development of endometriosis and fibroids. Exposure to phthalates during pregnancy and lactation may cause disorders of the reproductive system in offspring. Risk assessment for phthalate exposure presents an additional challenge because of the interactions between different pollutants and the complexity of interactions in the endocrine system [1,10,24,26,28,30,46,47]. Detailed information is provided in Table 4.

### 4.2. Bisphenols: Organic Chemicals That Belong to the Phenol Group

The most common is bisphenol A (BPA), which is used in the production of epoxy resins, polycarbonate plastic (PC), and other plastics and in the production of thermal paper (e.g., POS receipts). Plastics are used to produce food and drink packages, disposable products (e.g., plates and cups), microwave containers, children’s toys, plastic coatings (e.g., in food cans), water storage tools, and other daily use products. BPA may be detected in all these products and items. Bisphenol A is also used to produce food contact materials (FCMs), epoxy resin paints, printing ink, and flame retardants (FRs). Moreover, bisphenol A is used as an antioxidant in foodstuffs and cosmetics [1,8,10,35,36,41]. As shown in Table 2, the main sources through which humans come into contact with bisphenol A are foods and drinking water. Humans are also at risk of coming into contact with this compound through thermal paper, CDs, paints, glue, and electronic devices as well as through outdoor and indoor air in closed spaces due to the absorption of these chemicals in suspended dust. Bisphenol A enters the human organism through the digestive system, the breathing system, and the skin. It may be released from plastics and therefore may be present in foods, drinks, and baby food, especially if the packaging is warmed up, washed with detergent, or mechanically changed (pressed or extended). The main risk groups are infants and small children due to their low body weights and the ever more frequent use of plastic bottles, cups, plates, and spoons made from materials containing BPA. The European Food Safety Agency (EFSA) estimated that the total risk for infants and children of contacting BPA from food and non-food sources is slightly below the tolerable daily intake (TDI) of 4 μg × kg^−1^ bw × day^−1^. The risk was found to be lower in adults [9,29,34,35]. Exposure to BPA can have many health consequences, especially during the prenatal period. Numerous studies have confirmed that the presence of a high amount of BPA in the mother’s urine is associated with a lower body birth weight in the child. Additionally, changes in the reproductive systems of boys, changes in behavior (anxiety, depression, and increased aggression), and dysfunctions in thyroid function are frequently observed. For example, the exposure of pregnant women to BPA was shown to cause a decrease in the T4 concentration in women and a reduced TSH concentration in newborns. However, the authors indicated that these results were not statistically significant, but similar results have been obtained in various research centers [36,50]. A positive correlation between the concentration of BPA and obesity, which starts with an aggressive increase in body weight in the first 6 months of a child’s life, has been identified in both sexes [1,8,10,24,34,46,50]. Detailed information is presented in Table 5.

### 4.3. Parabens (PHB): A Group of Organic Chemicals Used as Preservatives in Cosmetics, Medicines, and Foods

Parabens are esters of para-hydroxybenzoic acid (PHBA) with different functional groups. They have fungistatic activity and weak antibacterial activity. The most widely used parabens are those with straight-chain alkyl groups, such as methylparaben (MP, E218), ethylparaben (EP, E214), propylparaben (PP, E216), butylparaben (BP), and heptylparaben (HP, E209). Less significant parabens are those with branched alkyl groups, such as isopropylparaben and isobutylparaben, as well as aryl groups, such as benzyl paraben. Sometimes, their salts are also used [1,38]. Due to their widespread use, humans are at risk from these chemicals through the consumption of foods whose production involves the use of preservatives from the paraben group. Risk also comes from skin contact, since parabens are commonly used in various cosmetics (e.g., soaps, shampoos, creams, etc.) [1,38,40]. Parabens do not bioaccumulate in the human organism; they are eliminated through the urine and feces [28,56]. Parabens are commonly considered to be chemicals that are ‘safe for human health and life’; however, the WHO classifies them as non-persistent EDCs, as they show agonistic activity towards estrogen receptors [1]. They may contribute to the development of breast cancer, inappropriate development of the male reproductive system during the fetal period, or unfavorable changes in the uterus [1,3]. They may also cause food and contact allergies as well as contact dermatitis [1,28,56].

### 4.4. Polycyclic Aromatic Hydrocarbons (PAHs): A Class of Organic Chemicals That Consist of Two or More Condensed Aromatic Rings

Polycyclic Aromatic Hydrocarbons develop as a result of incomplete thermal burning or through pyrolysis during the treatment of organic materials. They are complex chemicals that may consist of numerous combinations [1,11]. There are various sources through which people may be exposed to this group of chemicals. The main risk for non-smokers is processed foods, whereas for smokers, the risk from smoking is comparable to the risk from processed foods. Another health issue is exposure to PAHs while breathing, especially occupational exposure to PAHs (e.g., at waste incineration plants, crude oil refineries, and coke, asphalt, and aluminum production facilities) [1,39,57,58,59]. Foods may be contaminated by these chemicals by environmental sources as well as through food preservation methods (e.g., heating, drying, and smoking) and cooking processes (e.g., grilling and baking) [1,11,58,60]. Based on environmental monitoring studies, including those on foods, it was concluded that benzo-α-pyrene (BaP) may be used as a marker of exposure to the entire PAH group and to determine the margins of exposure (MOEs) for this group of compounds [11]. The WHO [1] classified benzo-α-pyrene as a chemical that has been proven to have endocrine-disrupting activities that affect human homeostasis (EDCs). The EFSA [11] found that the median risk for Europeans coming into contact with benzo-α-pyrene through food consumption is 235 ng × day^−1^ (3.9 ng × kg bw^−1^ × day^−1^). The main sources of PAHs are cereals, cereal products, and seafood (see Table 2). Epidemiological tests have concluded that PAHs have cancerogenic activity, e.g., contributing to lung cancer, as well as genotoxic and mutagenic activity, while BaP is a major promotor of pancreatic cancer and colorectal adenoma. Due to its affinity for fat, BaP is also detected in female milk, which puts babies at risk from the first days of their lives [1,38,60,61]. This is particularly important because of the influence of these chemicals on human reproductive activities. Their presence in the human organism can lead to male infertility due to competition for aryl hydrocarbon receptors (AhRs) [1,62]. In women, it leads to malfunction of the ovaries, which may result in premature menopause [61,63]. Detailed information is provided in Table 6.

## 5. Conclusions

The biomonitoring of xenoestrogens, which are chemicals with an unfavorable impact on human health, is a crucial tool that can be used to assess the risk caused by anthropogenic pollution from the group of endocrine disruptors. It is also an essential element in risk assessment for diseases of civilization. This mainly applies to chemicals with real or potential effects on the homeostasis of the human endocrine system (EDCs). This includes the chemicals discussed here and others, e.g., those classified as persistent organic pollutants (POPs). Numerous studies on the risks associated with cumulative and/or aggregate toxicity and potential interactions between these chemicals at the human body level have allowed the above hypotheses regarding possible adverse health effects due to their presence in human life to be formed. These findings also suggest that more intensive human actions should be conducted to eliminate these chemicals from the environment in order to minimalize their unwanted health consequences. This is particularly essential since humans are surrounded by a cocktail of anthropogenic chemicals that directly impact human health. In the era of fighting environmental pollution, in order to prevent its complete degradation, conducting so many studies related to the impact of EDCs on human health is a great positive contribution of the world of science to the precise determination of threats. With regard to these studies, it can only be noted that they focus solely on individual problems. What is new is a holistic approach to issues related to public health, consumer safety, and/or the environment, as well as the wide dissemination of research results in a manner accessible to the general public.

## Figures and Tables

**Table 1:** Levels of selected EDCs in human samples (ng × mL^−1^).

Country	BPA ^1^	ΣBP ^2^	PA ^3^	DEHP ^4^	PAH ^5^	Benzo-*α*-pyrene	Material	Parabens	References
EUROPE									[24,28,29,30,31]
Denmark	3.25	ND	274.20	ND	ND	ND	urine	ND	
Czech Republic	0.045	ND	ND	ND	ND	ND	serum	ND	
Poland	ND	ND	314.70	ND	ND	ND	urine	PBA 1.15	
Slovenia	1.55	ND	10.437	4.205	ND	ND	urine	ND	
The Netherlands	1.66	2.59	57.08	50.43	0.24	ND	urine	ND	
AMERICA									
US	1.65	1.90	569.50	281.90	0.10		urine	MP 32.05 PP 4.10 PBA 0.18	
Canada	1.20	ND	185.40	ND	0.18	<LOD	urine	ND	
ASIA									
India	5.08	5.12	ND	8.79 *	0.42	ND	semen urine	PBA 0.679	
Japan	0.84	2.02	124.11	ND	0.18	ND	urine	PBA 1.14	
China	1.10	1.33	232.35	ND	1.20	ND	urine	PBA 0.879	
Iran	ND	ND	ND	ND	0.36	ND	urine	ND	

All results are given as the median, * μg × mL^−1^; ^1^ BPA, bisphenol A; ^2^ BP, total bisphenol (sum of bisphenol A, bisphenol F, and bisphenol S); ^3^ PA, sum of phthalates; ^4^ DEHP, di(2-ethylhexyl) phthalate; ^5^ PAH, sum of polycyclic aromatic hydrocarbons; ND, not determined or no data available; LOD, limit of determination.

**Table 2:** Levels of selected EDCs in food (ng × g^−1^).

Products	BPA ^1^	ΣBP ^2^	PA ^3^	DEHP ^4^	PAH ^5^	Benzo-*α*-pyrene	Parabens	References
Meat and meat products	<LOD	0.07 **	8.47 **	5.16 **	2.11	0.32 (0.93/0.10) ^X^	30.22	[7,8,24,38,39]
Fish and fish products	0.23 **	2.02 **	<LOD	<LOD	13.82	1.30 (1.41/0.11) ^X^	0.53	
Dairy products	<LOD	0.05 **	<LOD	<LOD	0.84	0.13 (0.14/0.12) ^X^	39.45	
Vegetables	<LOD	<LOD	38.06 *	31.40	1.95	0.26 (0.15/0.32) ^X^	0.15	
Grains	<LOD	<LOD	ND	ND	3.08	0.40 (0.26/0.47) ^X^	95.88	
Infant and baby foods	<LOD	<LOD	ND	ND	0.41	0.05	ND	
Canned meat products	51.10	ND	ND	ND	ND	ND	ND	
Canned fish products	195.65	ND	ND	ND	ND	ND	ND	
Canned dairy products	15.20	ND	ND	ND	ND	ND	ND	
Canned vegetables	104.33	ND	ND	ND	ND	ND	ND	
Canned fruits	13.40	ND	ND	ND	ND	ND	ND	
Water (bottled and tap) (ng × L^−1^)	0.20	ND	ND	ND	1727.00	<LOD	ND	

All results are given as the median, * nmol × L^−1^, ** ng × mL^−1^. ^1^ BPA, bisphenol A; ^2^ BP, total bisphenol (sum of bisphenol A, bisphenol F, and bisphenol S); ^3^ PA, sum of phthalates; ^4^ DEHP, di(2-ethylhexyl) phthalate; ^5^ PAH, sum of polycyclic aromatic hydrocarbons; ^X^ (processed/unprocessed) food; ND, not determined or no data available; LOD, limit of determination.

**Table 3:** Levels of selected EDCs in different packaging materials (μg × g^−1^).

Material	BPA ^1^	ΣBP ^2^	PA ^3^	DEHP ^4^	Parabens	References
Glass	ND	ND	ND	ND	30.69	[38,40,41]
Cardboard	ND	ND	ND	ND	0.25	
Canned	ND	ND	ND	ND	9.68	
Plastic	ND	ND	ND	ND	97.29	
Paper	3.20 **	ND	ND	ND	1.96	
Others	ND	ND	ND	ND	36.02	
Dust	1000.00 *	2200.00 *	ND	ND	ND	
Personal care products ^5^	7.63 *	13.50 *	122.06	<LOD	ND	
Toys	0.14 ***	ND	ND	ND	ND	

All results are given as the median, * ng × g^−1^, ** ng × 100 g^−1^, *** ng × kg^−1^. ^1^ BPA, bisphenol A; ^2^ BP, total bisphenol (sum of bisphenol A, bisphenol F, and bisphenol S); ^3^ PA, sum of phthalates; ^4^ DEHP, di(2-ethylhexyl) phthalate; ^5^ Personal care products: body wash, hair care products, make-up, sanitary products, skin lotions, toilet soap, and toothpaste; ND, not determined or no data available; LOD, limit of determination.

**Table 4:** Impacts of selected phthalates on human health.

Names of the Substances	Main Sources of Exposure	Effects of Exposure	References
DEHP, MEP, MBP, MBzP, MEHP, MEHHP, MEOHP, MECPP, MiBP, MCPP, MnBP, MMP, ΣDEHP *	Inhalation (air), consumption (food), through the blood	Summary of phthalate metabolites associated with obesity, type 2 diabetes (related to decreased insulin secretion assessed by the fasting proinsulin-to-insulin ratio; exposure to phthalates induces mitochondrial dysfunction and, thus, oxidative stress, leading to the onset of insulin resistance), atherosclerosis, and hypertension (significant inverse correlation between MEP and both systolic (SBP) and diastolic blood pressure (DBP)).	[1,26,47,48]
DBP, DEHP, DEP, DIBP, DINP	Inhalation (air), consumption (food), through the skin, through the blood	The studies provided data on the association between phthalate exposure and cognitive performance. There is a suggestion of effect modification by sex, i.e., that girls may be more susceptible to cognitive effects of exposure than boys. Among studies with sex-specific results, girls had stronger associations in most studies.	[1,26,28,46,49]
BBP, DBP, DIBP	Inhalation (air), consumption (food), through the skin	Evidence for motor effects is stronger than for other outcomes, though uncertainty remains. For BBP, the evidence is moderate in girls, and a larger proportion of studies for DBP and DIBP indicated an inverse association than for other outcomes, though, for the latter two, there were also studies that reported positive (though non-significant) associations.	[1,26,28,47,49]
DEHP	Inhalation (air), consumption (food), through the blood	Studies reported an association between higher exposure and more frequent internalizing and externalizing problems. A single study on ADHD diagnosis with a medium level of confidence reported a significant association between summed metabolites and ADHD (OR: 1.5; 95% CI 1.1, 1.9). A study on social behaviors reported increased autistic behaviors with increased exposure to phthalates.	[1,26,30,47,49]
DEHP	Inhalation (air), consumption (food), through the blood	Reduced testis weight, diminished sperm count and quality (reduced mobility and increased percent of morphologically abnormal spermatozoa), increase in the frequency of defects of the reproductive system, and abnormalities in the sexual development of males, cryptorchidism in the offspring, and decreased anogenital distance.	[1,26,28,46,47]
BBP	Inhalation (air), consumption (food)	In men, reduced testis, epididymis, and prostate mass, and reduced sperm count with impaired quality (morphology). Increased frequency of abnormalities in the genitals of the offspring, decrease in the sperm cell count and sperm motility, increase in the percentage of abnormal sperm cells, disorders of testosterone production for adult males in the next generation, histopathological changes in the structure of the seminiferous tubules and Leydig cells, and delayed sexual puberty in the offspring.	[1,26,28,46]
DBP	Inhalation (air), consumption (food)	Disturbances in processes of differentiation and development of androgen-dependent tissues; hypoplasia or delayed development of the gonads; reduced testis and epididymis weights; pathological and biochemical changes in the testis, e.g., necrosis of seminiferous tubules; decreased reproductive ability; changes in the structure and function of the epididymis; hypospermia an increased percentage of abnormal spermatozoa; disturbances in the expression of genes that affect the development of androgen-dependent tissues; abnormal development of genitals; reduction of body and sexual organ weights; disturbances in the organogenesis of the testis; cryptorchidism; hypospadias; decreases in the count, viability, and motility of sperm; increases in the frequency of morphologically abnormal gametes and testosterone production in the offspring; disturbances in the metabolism of steroid hormones in the next generation; delayed sexual puberty; growth retardation; and disturbances in the sex ratio of the offspring.	[1,26,28,46]
NP	Inhalation (air), consumption (food), through the skin	Toxic effects in males, reduction of testis and epididymis weights, a decrease in sperm production, reduced viability and quality of gametes (reduced motility and an increase in the frequency of morphologically abnormal spermatozoa), damage to acrosomes, apoptosis of male gametes and Sertoli cells, increase in the frequency of breakage of DNA strands in haploid germ cells, and reduced production of testosterone.	[1,46]

* DEHP, di-2(2-ethylhexyl) phthalate; MEP, mono-ethylphthalate; MBP, mono-butyl phthalate; MBzP, mono-benzylphthalate; MEHP, mono-(2-ethylhexyl) phthalate; MEHHP, mono-(2-ethyl-5-hydroxyhexyl) phthalate; MEOHP, mono-(2-ethyl-5-oxohexyl) phthalate; MECPP, mono-(2-ethyl-5-carboxypentyl) phthalate; MiBP, mono-isobutyl phthalate; MCPP, mono-(3-carboxypropyl) phthalate; MnBP, mono-n-butyl phthalate; MMP, mono-methyl phthalate; ΣDEHP, di-2(2-ethylhexyl) phthalate metabolites.

**Table 5:** Impacts of selected bisphenols on human health.

Name of the Substance	Main Source of Exposure	Effects of Exposure	References
BPA	Inhalation (air, dust), consumption (food, drinking water, and FCMs), through the skin (care products, FCMs, and thermal paper)	High levels of human exposure can be associated with recurrent miscarriages. Exposure of pregnant women can also affect the frequency of premature deliveries.	[1,8,34,50,51]
BPA	Through the skin (baby care products and toys)	Baby care products containing di-2(2-ethylhexyl) phthalate (DEHP) could cause an increase in the total concentration of BPA.	[8,34,50]
BPA	Inhalation (air and dust), consumption (food, drinking water, and FCMs), through the skin (care products, FCMs, and thermal paper)	The birth weights of children with exposed mothers were significantly lower than those of children with unexposed mothers. The same was true for children whose fathers were exposed in comparison with children whose fathers were not exposed.	[1,8,34,50]
BPA	Inhalation (air and dust), consumption (food, drinking water, and FCMs), through the skin (care products, FCMs, and thermal paper)	Obesity and/or rapid body mass increase in infants during the first 6 months of life may be the result of maternal exposure during pregnancy.	[1,8,34,50]
BPA	Inhalation (air and dust), consumption (food, drinking water, and FCMs), through the skin (care products, FCMs, and thermal paper)	Exposure during pregnancy also has an impact on male genital development. Reduction of testis and epididymis weights, decrease in the male gamete count, increase in the frequency of abnormal spermatozoa, reduced sperm motility and sperm function, enhanced frequency of DNA strand breaks in haploid male germ cells, histopathological changes in the testes, and enhanced sensitivity of sexually immature animals.	[1,8,34,36,46,50,51,52]
BPA	Inhalation (air and dust), consumption (food, drinking water, and FCMs), through the skin (care products, FCMs, and thermal paper)	The concentration in the urine is also positively associated with asthma at the ages of 3, 5, and 7 years.	[1,8,34,50]
BPA	Inhalation (air and dust), consumption (food, drinking water, and FCMs), through the skin (care products, FCMs, and thermal paper)	Negative effects on the immune system have been shown in children aged over 6 years and adults. In children, a relationship between BPA concentration and high CMV (presence of cytomegalovirus) has also been observed.	[1,8,34,50]
BPA	Inhalation (air and dust), consumption (food, drinking water, and FCMs), through the skin (care products, FCMs, and thermal paper)	Exposure in utero may adversely affect thyroid function, especially in infants and youths. Proper functioning of the thyroid hormones during this period (in utero and in early childhood) is essential for normal neurological development; hence, the groups with the highest risk are pregnant women and infants. Exposure of women during pregnancy is associated with a reduction in the TSH level in infant boys and a decreased level of T4 in women during pregnancy.	[1,8,34,36,50]
BPA	Inhalation (air and dust), consumption (food, drinking water, and FCMs), through the skin (care products, FCMs, and thermal paper)	It has been shown that an increase in the concentration of BPA in the urine of mothers has a link with increased anxiety and depression and poorer emotional control in their children at the age of 3. Research suggests that the environmental exposure (median concentration in urine of 1.2 μg × L^−1^) of mothers during pregnancy may cause neurobehavioral effects in children. Several new studies have reported changes that may indicate effects on brain development (effect on neurogenesis and on gene expression, neuroendocrine effects, effects on the morphology of certain brain regions, etc.).	[1,8,34,50]
BPA	Inhalation (air and dust), consumption (food, drinking water, and FCMs), through the skin (care products, FCMs, and thermal paper)	May induce uncontrolled cell proliferation through the activation of several signaling pathways, including SRC1-3, c-RAF, and HER3 in breast cells and the AKT and GPER pathways in prostate cells as well as inducing mutations in the BRCA1 and two genes in the ovarian cells. Recently, several papers have highlighted either direct or indirect genotoxic effects in various in vitro tests.	[1,53,54]
BPA	Internal exposure	Exposure to environmental estrogens is recognized as a risk factor for the tumorigenesis of estrogen-dependent organs. Oestradiol-17β’s functions are mediated by estrogenic receptors (ERs) that are present in mammals in two nuclear forms: ER-*α* and ER-*β*. ERs function as ligand-induced transcription factors, activating the transcription of target genes through interactions with estrogenic response elements (ERE). As previously mentioned, BPA is a classic example of a synthetic estrogen and has both agonistic and antagonistic effects on ERs.	[1,54]
BPA	Internal exposure	Reduces apoptosis in cancer cells by inactivating proapoptotic proteins and activating survival proteins and anti-apoptotic signals. It can also cause cancer cells to escape the effects of anti-cancer drugs, leading to drug resistance, including changes in transporter expression, anti-apoptotic or proapoptotic genes, and/or prosurvival genes.	[1,54]
BPF, BPS	Inhalation (air and dust), consumption (food, drinking water, and FCMs), through the skin (care products, FCMs, and thermal paper)	Many studies indicate that the incidence of male reproductive function abnormalities (a decline in sperm quality) in humans has been increasing over the years, induce oxidative stress and biomacromolecule damage in human granulosa KGN cells.	[1,29,53,55]

**Table 6:** Impact of selected PAHs on human health.

Name of the Substance	Main Source of Exposure	Effects of Exposure	References
Benzo(*α*)pyrene (B-α-P)	Occupational exposure, tobacco smoking	PAHs are mutagenic human carcinogens. Their carcinogenicity is mostly caused by their ability to attach to DNA, thus generating several disorder effects that often lead to tumor initiation. Several epidemiological studies have incidentally shown an association between exposure and an increased risk of developing lung, skin, and bladder cancers and an increased risk of developing breast, kidney, prostate, larynx, blood (leukemia), brain, and colorectal cancers.	[11,29,59,63]
PAHs	Consumption of traditionally processed (smoked) foods	Adverse effects can sometimes manifest directly in the skeleton as, for example, poor bone quality, gross abnormalities, or reduced stature.	[11,29,39]
PAHs	Environmental exposure	Early life exposure has been linked with both fetal growth disruption and anemia, i.e., potential causes or factors of reduced skeletal size and porotic hyperostosis.	[11,29,39]
PAHs	Consumption of traditionally processed (smoked) foods, environmental exposure	PAHs induce lipid peroxidation and can disrupt the stability of cell and organelle membranes, leading to structural damage. Lipid peroxidation is a source of toxic lipid breakdown products (e.g., alkanes, alkenes, aldehydes, e.g., malondialdehyde, and alcohols) that irreversibly damage important cellular macromolecules in DNA and may be cytotoxic.	[1,11]
PAHs	Consumption of traditionally processed (smoked) foods, environmental exposure	They may also have an immunotoxic effect. During fetal life, there may be disturbances in the expression of surface cell markers as well as disorders in the normal course of maturation of immunocompetent cells.	[1,11]
PAHs	Consumption of traditionally processed (smoked) foods, environmental exposure	This is reflected in the development period in the form of cell suppression and humoral mechanisms of the immune response. The exposure causes changes in the organs of the immune system, which significantly affect its functions. Such changes appear in the bone marrow, thymus, spleen, and lymph nodes. Depending on the exposure conditions, decreases in the mass of the spleen and lymph nodes as well as thymic cortex atrophy were observed. In serum, decreases in the IgM and IgA immunoglobulin levels can be found as well as a significant decrease in NK (natural killer) cell activity in the spleen.	[1,11]
PAHs	Consumption of traditionally processed (smoked) foods, environmental exposure	PAHs can cause a decrease in the quality (reduced mobility, more abnormal forms) and amount of sperm produced as well as its ability to penetrate and fertilize the egg. In female reproductive cells and oocytes, an increase in the incidence of chromosome diploid anomalies has been observed.	[1,11]

## Data Availability

Not applicable because the work was based on the literature review.

## Abbreviations

Abbreviations used in this paper:

ADHD	attention-deficit hyperactivity disorder
AhRs	aryl hydrocarbon receptors
BaP	benzo-α-pyrene
BBP	benzylbutyl phthalate
BP	total bisphenol
BPA	bisphenol A
BPF	bisphenol F
BPS	bisphenol S
BP	butylparaben
CMV	presence of cytomegalovirus
DBP	di-n-butyl phthalate
DBP	diastolic blood pressure
DEHP	di-2(2-ethylhexyl) phthalate
DNA	deoxyribonucleic acid
E209	heptylparaben
E214	ethylparaben
E216	propylparaben
E218	methylparaben
EDCs	endocrine-disruptor chemicals
EFSA	European Food Safety Agency
ERE	estrogen response elements
ERs	estrogen receptors
FCMs	food contact materials
FRs	flame retardants
HQ	hazard quotient
HP	heptylparaben
IgA	immunoglobulin A
IgM	immunoglobulin M
MBP	mono-butyl phthalate
MBzP	mono-benzylphthalate
MEHP	mono-(2-ethylhexyl) phthalate
MEHHP	mono-(2-ethyl-5-hydroxyhexyl) phthalate
MECPP	mono-(2-ethyl-5-carboxypentyl) phthalate
MEOHP	mono-(2-ethyl-5-oxohexyl)
MEP	mono-ethylphthalate
MiBP	mono-isobutyl phthalate
MMP	mono-methyl phthalate
MiBP	mono-isobutyl phthalate
MnBP	mono-n-butyl phthalate
MOEs	margins of exposure
MP	methylparaben
NK	natural killer
NP	nonylphenol
PA	phthalates
PC	polycarbonate plastic
PAHs	polycyclic aromatic hydrocarbons
PBDEs	polybrominated diphenyl ethers
PCBs	polychlorinated biphenyls
PCV	polyvinyl chloride
PHB	paraben
PHBA	para-hydroxybenzoic acid
PoD	point of departure
POPs	persistent organic pollutants
PP	propylparaben
SBP	systolic blood pressure
T3	triiodothyronine
T4	thyroxine
TBT	tributyltin
TDI	tolerable daily intake
TSH	thyroid-stimulating hormone
